# Reply to Loh and Ren: Motivating action among climate change believers

**DOI:** 10.1073/pnas.2515426122

**Published:** 2025-09-03

**Authors:** Alyssa H. Sinclair, Danielle Cosme, Kirsten Lydic, Diego A. Reinero, Michael E. Mann, Emily B. Falk

**Affiliations:** ^a^Annenberg School for Communication, University of Pennsylvania, Philadelphia, PA 19104; ^b^Annenberg Public Policy Center, University of Pennsylvania, Philadelphia, PA 19104; ^c^Penn Center for Science, Sustainability, and the Media, University of Pennsylvania, Philadelphia, PA 19104; ^d^Department of Psychology, University of Pennsylvania, Philadelphia, PA 19104; ^e^Department of Earth and Environmental Sciences, University of Pennsylvania, Philadelphia, PA 19104; ^f^Wharton Marketing Department, University of Pennsylvania, Philadelphia, PA 19104; ^g^Wharton Operations, Information and Decisions Department, University of Pennsylvania, Philadelphia, PA 19104

A majority of people in the United States (73%) and globally (86%) believe that climate change is happening (1, 2), yet many are not acting on their beliefs (3). On a broad scale, mobilizing people who already believe in climate change is an important tactic and may be more effective than changing the minds of the minority [~14%, (1)] who deny climate change (4). Our intervention tournament identified several effective strategies to motivate action and information sharing among the majority of people who believe in climate change (5).

In their commentary (6), Loh and Ren advocate for considering selection bias before scaling interventions to reach broader audiences, and specifically note the possibility of backfire effects for climate change deniers.^*^ We appreciate the suggestion to investigate potential moderators and strategies to tailor interventions to different demographic groups. We share these goals and provide publicly available data to support exploration and follow-up studies.

We agree that checking for backfire effects is important before scaling interventions. In new exploratory analyses, we removed exclusion criteria related to belief in climate change and tested political ideology and belief as potential moderators. We found no evidence of backfire effects and no significant interactions with political ideology or belief (Fig. 1 and Table 1) (8). However, as relatively few participants denied climate change or reported very conservative ideology, additional samples would strengthen evidence. In recent follow-up studies, we have replicated effects of some interventions—without observing backfire—in nationally representative samples that were stratified by political ideology and included climate change deniers.

**Fig. 1.**
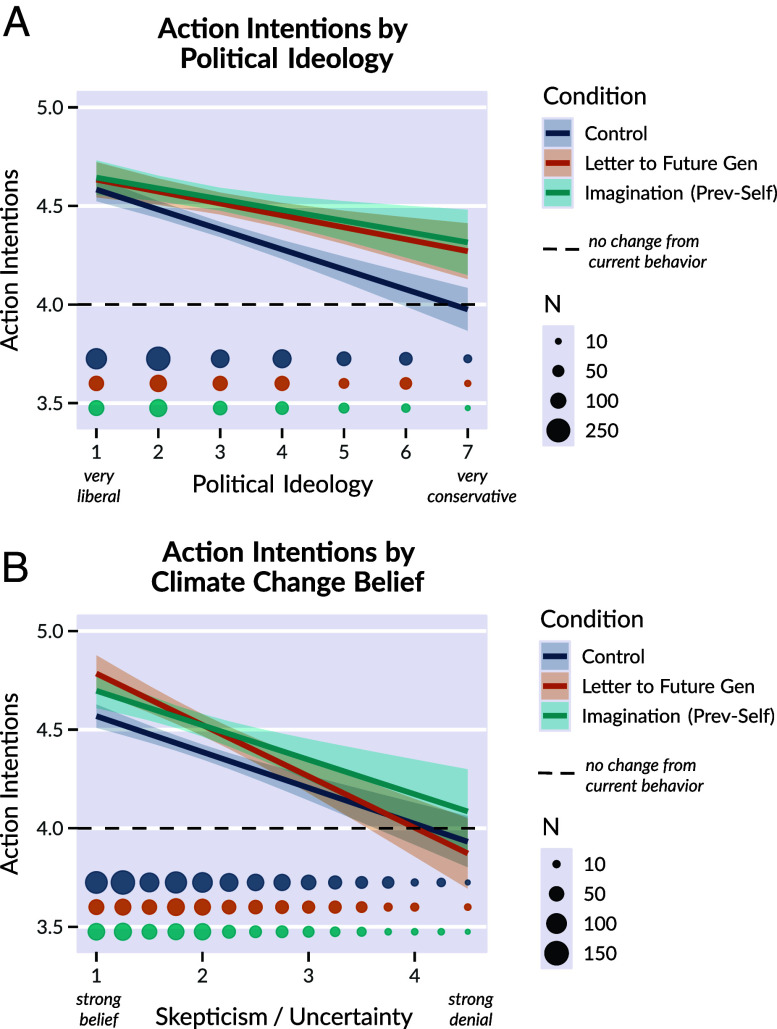
Effectiveness of leading interventions for increasing action intentions (*Letter to Future Generation* and *Guided Imagination, Prevention-Self*) across levels of political ideology (*A*) and climate change belief (*B*). Values below the dotted line indicate intention to engage in pro-environmental behaviors *less* often in the future. There were no significant backfire effects (values significantly below the dotted line or below the Control group) at any level of political ideology or belief. Numerically, the Intervention > Control effects for action intentions were stronger for conservatives than liberals (i.e., consistent with the idea of “room for growth” rather than backfire). Dot size reflects sample size per condition; note that given our recruitment approach, even without excluding climate change deniers post hoc, there were few participants who strongly disbelieved in climate change. Lines depict estimates from linear mixed effects regression models. Shaded bands indicate 95% CIs. Data and code are provided in an online repository (https://osf.io/x9c6j/).

**Table 1.** Pairwise contrasts (Intervention > Control) at fixed levels of political ideology and climate change belief

Contrast	β	SE	z-stat	*P*-value
A) Intervention effects at high levels of conservative ideology				
Action Planning (Collective) > Control	0.18	0.09	2.02	0.083
Action Planning (Individual) > Control	0.21	0.09	2.37	0.051
Carbon Footprint (General) > Control	0.03	0.09	0.34	0.824
Carbon Footprint (Personalized) > control	0.12	0.09	1.32	0.286
Imagination (Prevention-Other) > Control	0.09	0.09	1.01	0.406
Imagination (Prevention-Self) > Control	0.31	0.09	3.36	0.007
Imagination (Promotion-Other) > Control	0.09	0.09	0.95	0.418
Imagination (Promotion-Self) > Control	0.22	0.09	2.55	0.036
Impact Quiz > Control	0.19	0.09	2.26	0.054
Impact Text > Control	0.20	0.09	2.23	0.054
Letter to Future Gen > Control	0.27	0.08	3.23	0.007
Moral Values > Control	0.25	0.08	3.05	0.010
News Comments (Self-Rel) > Control	0.03	0.09	0.28	0.824
News Comments (Social-Rel) > Control	0.18	0.09	1.96	0.084
Personal Benefits > Control	0.27	0.08	3.31	0.007
Social Norms (Quiz) > Control	0.10	0.09	1.10	0.382
Social Norms (Text) > Control	0.01	0.08	0.07	0.941
B) Intervention effects at high levels of climate change uncertainty/skepticism				
Action Planning (Collective) > Control	0.03	0.09	0.28	0.884
Action Planning (Individual) > Control	−0.12	0.09	−1.27	0.691
Carbon Footprint (General) > Control	−0.03	0.09	−0.34	0.884
Carbon Footprint (Personalized) > control	−0.02	0.09	−0.28	0.884
Imagination (Prevention-Other) > Control	−0.15	0.10	−1.60	0.691
Imagination (Prevention-Self) > Control	0.14	0.09	1.43	0.691
Imagination (Promotion-Other) > Control	−0.12	0.09	−1.36	0.691
Imagination (Promotion-Self) > Control	0.02	0.09	0.24	0.884
Impact Quiz > Control	0.02	0.09	0.25	0.884
Impact Text > Control	0.13	0.09	1.53	0.691
Letter to Future Gen > Control	−0.02	0.08	−0.21	0.884
Moral Values > Control	−0.05	0.09	−0.63	0.884
News Comments (Self-Rel) > Control	−0.09	0.09	−0.94	0.855
News Comments (Social-Rel) > Control	0.01	0.09	0.12	0.908
Personal Benefits > Control	−0.06	0.09	−0.75	0.855
Social Norms (Quiz) > Control	−0.07	0.09	−0.77	0.855
Social Norms (Text) > Control	−0.06	0.08	−0.77	0.855

Omnibus tests revealed no significant interactions that would suggest that effects of ideology or belief on action intentions differed across conditions; however, we report follow-up tests for transparency. A) Intervention effects for “very conservative” ideology (rating of 7 on a 7-pt scale; z = 2.55). For several interventions (green highlights), action intentions were greater than Control, similar to the effects observed across all levels of political ideology. No interventions showed significant backfire effects (Control>Intervention). B) Intervention effects at a high level of uncertainty/skepticism indicating disbelief in climate change (mean score of 4; z = 2.7). No interventions showed significant backfire effects (Control > Intervention). Estimates are false-discovery-rate corrected. Data and code are provided in an online repository (https://osf.io/x9c6j/). Overall, results suggest that intervention effectiveness may numerically differ across demographic groups, but we do not observe evidence of backfire that would undermine broad implementation.

Nonetheless, as stated in our paper, we agree that “an important goal for future research is to identify strategies that are effective for individuals who hold doubtful or dismissive beliefs related to climate change” (5). We are also aligned with the goal of tailoring intervention strategies to relevant subpopulations. For example, Loh and Ren’s reanalysis is related to literature on addressing disparities in health communication across socioeconomic status (SES) and a broader need for message tailoring and targeting for relevant audiences (9).

Beyond the present study, we agree that it is important to recruit representative samples, explore strategies to overcome political partisanship, and investigate how interventions can be tailored to different audiences. In ongoing work, we are also exploring how other factors, particularly SES and age, moderate intervention effectiveness. A tournament approach enables researchers to compare effectiveness across strategies and audiences. We support further research that tailors interventions to different demographic groups, while also proposing that our leading interventions show promise and are worth testing in the field to assess scalability and impact.

## Footnotes

^*^Loh and Ren conducted a sensitivity analysis with our publicly available data, but opted to use SES, age, gender, and education as proxies for belief in climate change. These demographic variables are weaker predictors of climate change belief relative to other metrics, like political orientation (7). Notably, direct measures of belief are included in our public dataset, along with political ideology/affiliation variables. We encourage researchers to contact the corresponding authors with questions about the data.
